# Research status of subclinical hypothyroidism promoting the development and progression of cardiovascular diseases

**DOI:** 10.3389/fcvm.2025.1527271

**Published:** 2025-04-04

**Authors:** Peijie Wang, Weiming Zhang, Haiyan Liu

**Affiliations:** ^1^ Department of Nuclear Medicine, First Hospital of Shanxi Medical University, Taiyuan, Shanxi, China; ^2^ Collaborative Innovation Center for Molecular Imaging of Precision Medicine, Shanxi Medical University, Taiyuan, Shanxi, China

**Keywords:** subclinical hypothyroidism, cardiovascular disease, lipid disorders, atherosclerosis, diastolic heart function, levothyroxine, TSH

## Abstract

In recent years, the incidence of cardiovascular disease (CVD) has risen steadily, significantly impacting public health. Subclinical hypothyroidism (SCH) remains a controversial risk factor for CVD. This review examines the associations between SCH and dyslipidemia, carotid intima-media thickness (C-IMT), cardiac dysfunction, and cardiovascular event risk. Evidence suggests SCH may exacerbate atherosclerosis and cardiac dysfunction through mechanisms such as increased LDL synthesis, oxidative stress, and impaired vascular endothelial function. However, the causal link between SCH and cardiovascular outcomes remains unclear due to study design heterogeneity and overreliance on TSH levels. Elevated TSH may not solely reflect thyroid dysfunction but could also indicate compensatory responses to inflammation, aging, or stress. Large-scale studies like NHANES and IPD meta-analyses show a strong association between SCH and cardiovascular risk in younger populations, which diminishes in older adults due to physiological TSH increases. The cardiovascular benefits of levothyroxine (L-T4) therapy in SCH patients are limited, especially in older individuals, where a narrow therapeutic window increases side effect risks. Studies relying solely on TSH as a diagnostic and therapeutic target have significant limitations, as TSH cannot distinguish adaptive thyroid adjustments from pathological states and overlooks the role of free thyroid hormones (FT3/FT4). Future research should integrate multi-dimensional markers (such as oxidative stress indicators, vascular elasticity measures, and thyroid antibody status) and adopt longitudinal study designs to more accurately assess the clinical significance of SCH.

## 1 Introduction

Subclinical hypothyroidism (SCH) is a metabolic condition characterized by mild or no symptoms, defined by normal free thyroxine (FT4) levels and elevated thyroid-stimulating hormone (TSH) levels, indicating subtle thyroid dysfunction (1). SCH is often categorized into mild and severe forms based on TSH levels, with a cutoff of ≥10 mIU/L (2). However, classifications may vary due to age and disease status in different populations. In elderly populations, TSH levels naturally increase with age due to a decline in hypothalamic-pituitary-thyroid (HPT) axis function, often leading to a compensatory slight increase in TSH secretion. Consequently, L-T4 treatment may not significantly benefit these patients (3).

Key factors contributing to the development of SCH include thyroid tissue damage, intake of goitrogenic substances like iodide, lithium, and antithyroid drugs, as well as autoimmune conditions such as thyroiditis (4). TSH, primarily released by the anterior pituitary gland under the influence of thyrotropin-releasing hormone (TRH) from the hypothalamus, stimulates the synthesis and secretion of thyroid hormones, primarily T4 and T3. The secretion of TSH is regulated by feedback from circulating thyroid hormones. Decreased thyroid hormone levels, as in primary hypothyroidism, trigger increased TSH release, while elevated levels suppress further TSH secretion (5–7). However, an elevated TSH concentration does not always indicate early thyroid dysfunction. It may reflect an increased set point of homeostasis in the HPT axis. Factors such as bacterial infections, systemic inflammation, drastic environmental changes, or chronic psychosocial stress (Post-traumatic stress disorder,PTSD) can shift the body's homeostatic set point, leading to higher TSH levels despite unchanged FT4 levels. Elevated TSH levels may thus result from factors beyond SCH (8, 9), and may be associated with an increased risk of cardiovascular issues. This article reviews the latest research on the relationship between SCH and CVD, aiming to enhance prevention and management strategies. A comprehensive review of English-language literature published since 2000, extracted from the PubMed/Medline database, is provided. Search terms included “subclinical hypothyroidism”, “cardiovascular disease”, “lipid disorders”, “atherosclerosis”, “diastolic heart function”, “levothyroxine”, and “TSH”.

## 2 Dyslipidemia in patients with subclinical hypothyroidism

### 2.1 Increased levels of low-density lipoprotein

3-Hydroxy-3-methylglutaryl-CoA reductase (HMGCR), the rate-limiting enzyme in cholesterol biosynthesis, is regulated by its phosphorylation state, with phosphorylation inactivating and dephosphorylation restoring its activity. As the pivotal regulatory step in the cholesterol synthesis pathway, HMGCR activity directly influences pathway flux and correlates positively with cholesterol production (10). TH enhances cholesterol synthesis by stimulating the transcription of the low-density lipoprotein receptor (LDL-R) gene, thereby elevating hepatic HMGCR expression (11). AMP-activated protein kinase (AMPK), known as the “metabolite-sensitive kinase”, plays a critical role in phosphorylating HMGCR. When TSH binds to the TSH receptor (TSHR) on hepatocytes, it activates the cAMP/PKA signaling pathway, leading to PKA-mediated phosphorylation of AMPK's inhibitory site. This reduces AMPK activity in hepatocytes, increasing HMGCR expression and promoting cholesterol synthesis, which contributes to dyslipidemia (12).

Proprotein convertase subtilisin/kexin type 9 (PCSK9) is a serine protease that interacts with the LDL-R on liver cells. LDL-R plays a crucial role in clearing LDL particles from the bloodstream. Upon binding to LDL, LDL-R internalizes and degrades these particles, releasing free cholesterol. PCSK9 enhances the lysosomal degradation of LDL-R, reducing its levels and impairing LDL clearance, thereby increasing low-density lipoprotein cholesterol (LDL-C) in the blood. Elevated LDL-C contributes to hyperlipidemia and is associated with dyslipidemia and inflammatory responses, increasing cardiovascular risk (13). A population-based cross-sectional study examined 1,003 middle-aged individuals, including those with normal thyroid function and SCH, excluding those on thyroid medication or with a history of metabolic disorders. SCH patients had higher serum TSH levels but normal FT3 and FT4 levels, ensuring the study was not confounded by thyroid hormones. The findings indicated elevated serum PCSK9 levels in SCH patients compared to those with normal TSH, suggesting a possible link. An *in vitro* experiment further explored TSH's effect on PCSK9 expression in hepatocytes. Elevated TSH bound to the TSHR, activated adenylyl cyclase (AC), increased intracellular cAMP, and triggered the cAMP-PKA-CREB pathway, leading to increased PCSK9 expression, synthesis, and secretion (14). However, the study's limitations included the inability to directly correlate *in vitro* TSH concentrations with human serum levels and the lack of subgroup analysis based on varying TSH levels. Future research should stratify participants by serum TSH levels for a more robust analysis.

In a descriptive cross-sectional retrospective analysis, researchers excluded individuals with low T3 and FT4 levels and randomly selected 447 patients without hypothyroidism history or prior treatment. The study assessed factors including gender, age, lipid profiles, and TSH levels. Results showed higher LDL levels in SCH patients compared to those without SCH (15). However, lifestyle and genetic factors contributing to lipid abnormalities were not considered. Future research should explore these variables to better understand the interplay between thyroid function and lipid metabolism. Elevated LDL levels promote lipid accumulation in arterial walls, increasing atherosclerosis and cardiovascular risk (16). SREBP2 (Sterol Regulatory Element-Binding Protein Transcription Factor 2) plays a pivotal role in cellular cholesterol synthesis and LDL-R gene regulation. It can enhance PCSK9 expression, and TSH levels positively correlate with circulating PCSK9, possibly mediated by SREBP2 (17). HDL (High-Density Lipoprotein) is renowned for its cardio-protective effects, attributed to its anti-inflammatory, antioxidant properties, endothelial enhancement, and lipid metabolism regulation. A cross-sectional study on SCH patients, excluding those with overt thyroid or other medical issues and not on thyroid medication, revealed a link between SCH and reduced levels of small HDL particles (sHDL-P). This association persists even after considering factors like smoking, weight, and gender, suggesting a potential tie to increased cardiovascular risk, as smaller HDL particles are believed to provide stronger cardiovascular protection (18).

### 2.2 Lipid peroxidation

8-iso-Prostaglandin F2α (8-iso-PGF2α) is a biomarker indicating oxidative stress, which contributes to atherosclerosis via vascular inflammation, lipid deposition, and plaque instability. Therefore, it's a valuable indicator for assessing atherosclerotic risk. A study on 40 thyroidectomy patients revealed that at the onset of subclinical hypothyroidism, 8-iso-PGF2α levels paralleled changes in TSH. Following two months of LT4 replacement therapy, changes in 8-iso-PGF2αcontinued to positively correlate with TSH changes. Even after adjusting for thyroid hormone levels and blood pressure, this relationship persisted. This suggests that elevated TSH may enhance lipid peroxidation, potentially promoting platelet activation related to atherosclerosis (19). However, the study's small sample size limits its generalizability, necessitating further research with larger cohorts. Additionally, since the experiment did not include a placebo group, it cannot fully rule out the possibility that reductions in 8-iso-PGF2α levels may occur independently of the replacement therapy. Hydroxyoctadecadienoic acid (HODEs) and hydroxyeicosatetraenoic acid (HETEs) are stable end products of lipid peroxidation. A study on lipid peroxidation, which diagnosed SCH using two measurements over six months to exclude laboratory errors or transient elevations, included 129 patients. Participants were categorized into normal, mild SCH (4.5 ≤ TSH <10 mU/L), and severe SCH (TSH ≥10 mU/L) groups based on their TSH levels. There were no significant differences in demographic and clinical factors, including age, gender, BMI, blood pressure, fasting glucose, total cholesterol (TC), HDL-C, and triglycerides (TG) at baseline. The results showed significantly elevated levels of HODEs and HETEs in both mild and severe SCH patients compared to the normal group (20). This indicates that SCH may intensify oxidative stress, potentially increasing the risk of atherosclerosis. Although the sample sizes were limited, these studies provide valuable initial insights. Future research should involve larger cohorts to validate the findings and offer more reliable clinical guidance.

### 2.3 Carotid intima-media thickened

Carotid intima-media thickness (C-IMT) reflects inflammation, lipid deposition, and fibrous tissue proliferation in the vascular wall. It is widely recognized as an early indicator of atherosclerosis and a significant predictor of coronary artery and cerebrovascular diseases. Elevated TSH levels are associated with lipid metabolic disorders and oxidative stress, particularly increased LDL-C and triglycerides, which contribute to increased C-IMT. A retrospective study involving 100 women aged 30–70 compared a SCH group with a normal thyroid function (control) group. Exclusion criteria included FT4 values outside the reference range or a history of thyroid disease. B-mode ultrasound was used to measure C-IMT, and correlations with serum FT4, TSH, C-reactive protein (CRP), and lipid levels were analyzed. Age showed the strongest correlation with C-IMT, and multivariate analysis revealed significant correlations with both age and TSH levels. SCH patients exhibited higher TSH levels, significantly increased C-IMT, and greater plaque formation frequency, with TSH levels positively correlating with C-IMT values (21). In a cross-sectional analysis of 8,623 participants in Brazil, a positive correlation was observed between mean C-IMT and SCH after adjusting for age, sex, race, and cardiovascular risk factors. Although the correlation was weak, mean C-IMT values were significantly higher in SCH participants compared to those with normal thyroid function (22). Despite the study's large scale, its cross-sectional design limits causal inferences, though elevated lipid levels may act as mediators influencing intima-media thickness (IMT) values. Yao et al. conducted a meta-analysis of 27 studies involving 1,065 SCH cases and 866 controls, finding that 494 SCH patients had significantly higher C-IMT compared to 390 controls (23). This finding is supported by other studies (24), further emphasizing the association between subclinical thyroid dysfunction and elevated carotid intima-media thickness.

## 3 Cardiac dysfunction in patients with subclinical hypothyroidism

### 3.1 Impaired ventricular diastolic and systolic function

The contraction function of the myocardium is intricately regulated by sarcoplasmic reticulum calcium adenosine triphosphatase (SERCA2), which pumps calcium ions back into the sarcoplasmic reticulum to maintain normal cardiac contraction and relaxation. Previous research has shown that T3 enhances myocardial contractility by upregulating SERCA2 expression (25). Conversely, TSH binding to the TSHR on cardiomyocyte membranes activates the downstream cAMP-PKA signaling pathway, leading to PKA phosphorylation of SERCA2a protein. This phosphorylation may inhibit SERCA2a activity, disrupting calcium ion homeostasis in cardiomyocytes, which can impair ventricular diastolic and systolic function and potentially contribute to ventricular remodeling (26). Additionally, a study on peak filling rate (PFR) provided similar insights. PFR reflects the maximum rate of blood filling the ventricles during diastole, indicating early diastolic filling capacity. The study included 36 participants: 20 patients with Hashimoto's thyroiditis who met SCH criteria, with thyroid function reassessed after a month to minimize laboratory errors and transient abnormalities, and 16 controls. The researchers found that patients with TSH levels ≥10 μIU/ml had significantly reduced PFR, indicating impaired diastolic function (27). This further confirms the negative impact of high TSH levels on cardiac function. However, due to the small sample size, larger studies are needed to validate these findings.

### 3.2 Aortic stiffness increased

Aortic stiffness is a critical predictor of morbidity and mortality in CVD, primarily due to age-related vascular stiffness caused by aging and degradation of elastic fibers in the arterial wall. Pulse wave velocity (PWV) reflects the speed at which the pulse pressure wave travels through blood vessels, while vascular compliance measures the vessel wall's ability to stretch in response to pressure changes. Lower compliance indicates reduced vessel elasticity, which increases resistance to blood flow and pulse wave propagation, thereby elevating PWV. Thus, increased aortic stiffness is associated with decreased compliance and higher PWV. Stella Bernardi et al. reviewed 11 observational studies and found that both subclinical and overt hypothyroidism patients exhibited significantly higher PWV compared to controls. Subgroup analysis revealed no significant difference in PWV between subclinical and overt hypothyroidism patients, suggesting that elevated PWV may increase cardiovascular risk in both groups (28). However, given the small-scale and non-randomized nature of most studies included in the meta-analysis, as well as differences in participant demographics and PWV measurement sites, these findings require further validation. Flow-mediated dilation (FMD) assesses the change in brachial artery diameter following a brief interruption and restoration of blood flow. Upon restoration, endothelial cells respond to changes in blood flow shear stress by releasing nitric oxide (NO), causing vasodilation and increased arterial diameter. Another study indicates that elevated PWV coupled with reduced FMD may signify endothelial dysfunction, where endothelial cells fail to respond effectively to shear stress, leading to diminished NO release and reduced vasodilation, thereby increasing cardiovascular risk (23).

### 3.3 Increased blood viscosity

A shortened activated partial thromboplastin time (APTT) suggests enhanced blood clotting ability, potentially altering blood rheological properties. The study included 50 controls and 141 untreated patients (116 females and 25 males). Based on pre-study TSH and free FT4 levels, patients were categorized into three subgroups: SCH with elevated TSH but normal thyroid hormones, moderate hypothyroidism with TSH ≤50 mU/L, and severe hypothyroidism with TSH >50 mU/L. Compared to the control group, patients with SCH exhibited significantly shortened APTT values and increased fibrinogen (FIB) levels, indicating a hypercoagulable state and an increased risk of thrombosis (29). The exact mechanisms warrant further exploration, but increased fibrinogen levels may contribute by altering blood rheological properties and enhancing plasma viscosity. APTT and fibrinogen have synergistic effects on thrombus formation. Another study arrived at similar conclusions, suggesting that changes in thyroid hormone levels in SCH patients affect liver metabolic functions, leading to dyslipidemia. This study found higher levels of Factor VII (FVII) and tissue-type plasminogen activator (t-PA), along with significantly reduced fibrinolytic capacity in SCH patients. These findings suggest that SCH may have a net prothrombotic effect (30).

### 3.4 Reduced carbon monoxide levels

**Figure 1:**
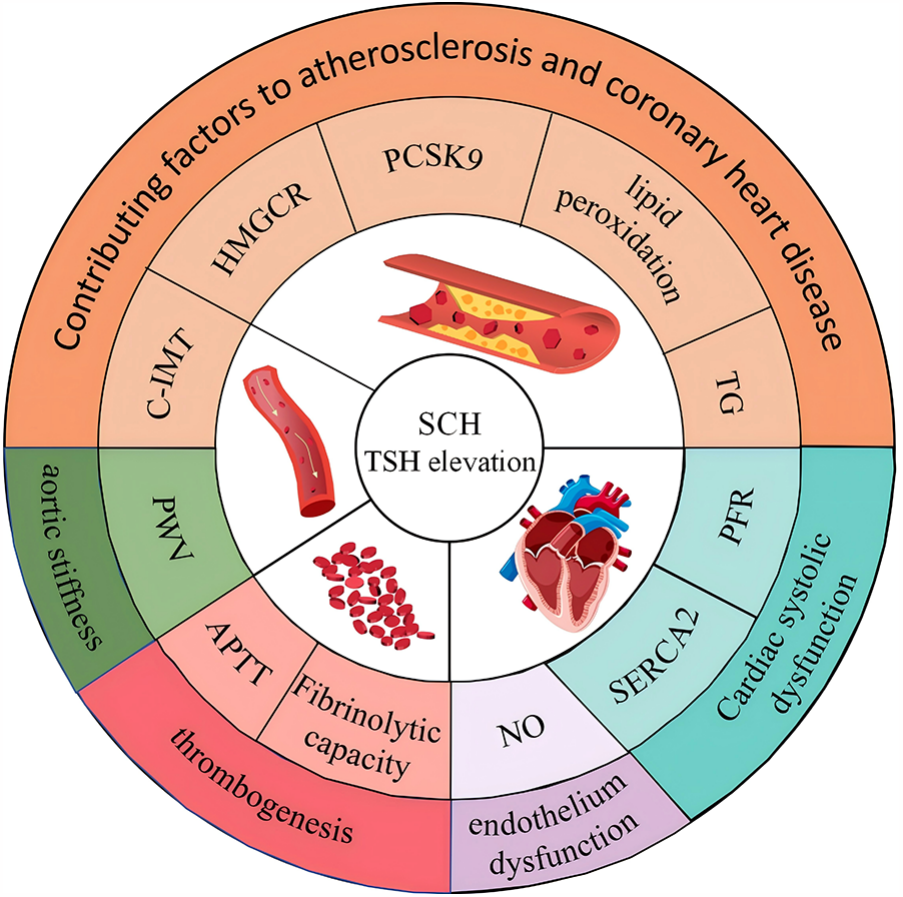
Impact of elevated TSH in subclinical hypothyroidism on lipid metabolism and cardiac function.

NO is primarily synthesized in vascular endothelial cells by nitric oxide synthase (NOS), an enzyme family with three subtypes, of which endothelial nitric oxide synthase (eNOS) is the main physiologically active form (31). NO activates soluble guanylate cyclase (sGC), increasing cyclic guanosine monophosphate (cGMP) levels, which in turn activates protein kinase G (PKG) and its downstream signaling pathways. This process decreases intracellular calcium ion concentration, leading to smooth muscle relaxation and regulation of vascular tone (32). In an experiment with 60 male rats, animals were randomly divided into three groups: a normal thyroid group, a SCH group, and a thyroid hormone treatment (SCH + T4) group, each consisting of 20 rats. The SCH model was induced by gavage, while the SCH + T4 group received L-T4 treatment at specific intervals, and the control group received physiological saline. Results showed that NO levels were reduced in the SCH group. However, in the SCH + T4 group, TSH concentrations decreased to levels similar to the control group, and NO levels increased (33). According to the discussion, elevated TSH levels in SCH may promote vascular smooth muscle cell proliferation and endothelial dysfunction, thereby affecting NO production and release, leading to reduced NO levels. Another study supports this, noting that SCH decreases nitric oxide utilization efficiency (34), though its clinical significance remains unclear. In summary, even with normal thyroid hormone levels, patients with SCH may be at risk for CVD due to elevated TSH levels, which can affect lipid metabolism and cardiac function (see Figure 1).

## 4 The incidence of cardiovascular disease in subclinical hypothyroidism

Thyroid hormones (TH) and TSH play a critical role in cardiovascular health, influencing the entire circulatory system. A comprehensive meta-analysis of 555,530 participants across 32 prospective cohort studies revealed that SCH is moderately associated with increased risk of CVD and higher all-cause mortality compared to individuals with normal thyroid function. Subgroup analyses stratified by CVD risk demonstrated that SCH significantly elevates CVD and all-cause mortality risks in the high CVD risk subgroup, with relative risks (RR) of 2.20 (95% CI: 1.28–3.77) and 1.66 (95% CI: 1.41–1.94), respectively. Conversely, in the low CVD risk subgroup, SCH was moderately associated with CVD risk but showed no notable association with all-cause mortality. Age-based subgroup analyses revealed that in participants under 65 years, SCH was linked to increased CVD risk and all-cause mortality, with RRs of 1.54 (95% CI: 1.21–1.96) and 1.28 (95% CI: 1.10–1.48), respectively. However, this association was not evident in studies involving participants aged 65 years or older. Further analysis in older adults (≥65 years) showed that SCH was not significantly associated with CVD risk or all-cause mortality in the low CVD risk subgroup. In the high CVD risk subgroup, SCH was associated with elevated all-cause mortality, though the result was not statistically significant, likely due to the physiological increase in TSH levels with age (35). This meta-analysis highlights the importance of identifying SCH patients who may benefit from targeted treatment based on their CVD risk. However, it is crucial to acknowledge the limitations of applying population-level data to individual cases (36), as most studies measured TSH and T4 only once at baseline. Future clinical practice should consider individual etiological variations when assessing the health risks of SCH patients. This nuanced understanding will enhance personalized approaches to managing potential cardiovascular complications in this population. Other studies focusing on the elderly and systematic reviews have also produced similar results (37, 38). Moreover, systematic reviews indicate that individuals with higher TSH levels, especially those with SCH and TSH concentrations ≥10 mIU/L, have a significantly increased risk of coronary heart disease (CHD) events and CHD-related mortality (39).

Another study based on the U.S. National Health and Nutrition Examination Survey (NHANES) involving 7,626 adults aged 20 years and older provided contrasting insights (40). By analyzing TSH, FT4, and FT3 levels along with mortality data and employing a Cox proportional hazards model, the study assessed the relationship between HPT axis hormones and lifespan. The findings indicated that HPT axis function declines with age, marked by elevated FT4 and decreasing FT3 levels. This decline may result from reduced deiodinase enzyme efficiency in converting T4 to T3 or variations in the thyroid's T4/T3 production ratio. Notably, higher FT3 levels were associated with lower mortality, while higher FT4 levels correlated with increased mortality, suggesting that declines in T3 and the T3/T4 ratio significantly influence lifespan. Non-parametric analysis further revealed that FT3 levels declined more sharply with age compared to FT4 and TSH. These hormonal changes aligned with mortality findings, emphasizing their role in lifespan regulation. After adjusting for disease-related covariates and inflammatory markers, the results remained consistent. This underscores the potential of therapies targeting the T3/T4 ratio, particularly for elderly patients on levothyroxine treatment. Expanding the study sample to include diverse racial and regional populations is also essential to validate these findings. Additionally, the study highlights FT3 as a principal effector molecule, recommending enhanced clinical focus on free T3 levels for improved thyroid disorder diagnosis and management.

A study using individual participant data (IPD) from 11 prospective cohort studies investigated the relationship between thyroid function and atrial fibrillation (AF) risk, involving 30,085 individuals. After adjusting for age and gender, no association was found between TSH levels within the normal range and AF risk. Continuous TSH analysis revealed a slightly increased AF risk at lower normal TSH levels and a slight decrease at higher levels, though the hazard ratio (HR) remained near 1.0. SCH, regardless of severity, showed no association with AF occurrence after adjusting for age and gender. Baseline FT4 levels were positively associated with AF risk, with higher FT4 levels linked to increased risk, a finding consistent across multivariate adjustments and additional factors. However, in participants with a history of CVD, FT4 levels were not associated with AF risk (41). Although the study had a large sample size, the majority of participants were white and relatively young, limiting generalizability. Future research should include diverse ethnic and age groups and assess changes in thyroid function over time to provide new insights into AF risk assessment.

## 5 Treatment of subclinical hypothyroidism

### 5.1 Real effects of levothyroxine therapy

Levothyroxine (L-T4), a standard hypothyroidism treatment, has demonstrated a capacity to reduce C-IMT, potentially preventing or slowing atherosclerosis. This beneficial effect is primarily attributed to its impact on key lipid parameters, including total cholesterol, triglycerides, LDL, blood pressure, and lipoprotein levels. Specifically, thyroid hormones can enhance eNOS expression and activity, leading to increased NO production and lower LDL-C levels (42). L-T4 therapy may also reduce the risk of heart failure and enhance cardiac function through various mechanisms, including improved cardiac output, vasodilation, and anti-inflammatory and antioxidant pathways (43). However, it's important to note that for patients with SCH, the treatment outcomes may not be as favorable. Significant therapeutic effects from L-T4 are primarily observed in cases of overt hypothyroidism. A comprehensive meta-analysis, encompassing 58 high-quality studies with diverse populations and genders, was conducted to explore the associations between thyroid hormone levels and various clinical parameters. The analysis revealed a consistent trend across the studies, with a total of 1880 association analyses. In particular, the study compared the frequencies of associations between thyroid hormones (FT4, TT3/FT3, TSH) and clinical outcomes such as atrial fibrillation, cardiac measurements, osteoporosis, and fractures. The findings indicated that thyroid hormone levels, especially FT4, exhibited stronger associations with clinical parameters compared to TSH levels. The strong negative correlation between thyroid hormone levels and TSH largely explained the relationship between TSH and clinical outcomes. Consequently, the current clinical and research practices that rely primarily on TSH levels for thyroid function assessment may require reevaluation. Categorizing thyroid status based on thyroid hormone levels, particularly FT4, appears to be a more robust approach both theoretically and empirically (44).

His study evaluated the effects of L-T4 therapy in 117 patients with SCH and heart failure with reduced ejection fraction (HFrEF), divided into an experimental group (L-T4 combined with standard HFrEF treatment) and a control group (standard HFrEF treatment alone). Patients ranged in age from 42 to 72 years. Thyroid function tests were performed within 24 h of hospitalization, with a second test conducted 10–14 days after HFrEF diagnosis to confirm SCH. No significant differences in baseline characteristics were found between the two groups. The primary endpoint, assessed using the 6-Minute Walk Test (6MWT), evaluated the efficacy of L-T4 treatment. Thyroid function was reassessed at weeks 4, 8, 12, and 24 to guide L-T4 dose adjustments. After 24 weeks, the experimental group showed a significant reduction in TSH [from 6.23 (5.30, 7.92) to 4.07 (2.56, 5.12) mIU/L] compared to the control group. However, the experimental group also exhibited significantly higher FT3 [from 2.64 (2.31, 3.02) to 2.99 (2.73, 3.81) pg/ml] and TT4 [from 7.90 (6.43, 8.68) to 8.20 (7.30, 9.10) μg/dl]. Both groups improved in 6MWT distance, but the experimental group showed greater improvement after adjusting for age, sex, left ventricular ejection fraction (LVEF), TSH, and hypertension history. Subgroup analyses revealed that benefits were primarily observed in men, patients under 65 years, and those with TSH >6.52 mIU/L, with no significant effects in patients over 65 years (45). The findings suggest that L-T4 therapy may be a promising adjunct for HFrEF patients with SCH. However, while L-T4 effectively reduced TSH, it also elevated FT3 and TT4 levels, potentially leading to thyroid hormone dysregulation. Given that thyroid hormone levels, particularly FT4, are more clinically relevant than TSH, the observed increases may complicate thyroid function management in these patients. Future studies should explore these effects further, particularly in different patient subgroups.

A clinical trial divided 95 patients with acute myocardial infarction and persistent mild SCH into a L-T4 group and a placebo group. Participants' ages ranged from 62.9 to 64.1 years. TSH and thyroid hormone levels were measured twice at 7- to 10-day intervals to confirm SCH. The L-T4 group had TSH levels monitored at weeks 4, 8, 12, and 24 (±7 days), with dosage adjustments based on results. Both groups showed improvements in left ventricular ejection fraction (LVEF) after 52 weeks. However, there was no significant difference in LVEF improvement between the L-T4 and placebo groups. Serum TSH levels decreased in both groups over 52 weeks, with the L-T4 group exhibiting significantly lower median TSH [1.8 mU/L (interquartile range, 1.3–2.2)] compared to the placebo group [3.2 mU/L (interquartile range, 2.7–4.2)]. FT4 levels were also higher in the L-T4 group (46). The decrease in TSH in the placebo group was not explicitly explained but may be attributed to stress from acute myocardial infarction, which could disrupt HPT axis function, reducing TSH secretion. Alternatively, cardiovascular medications used during treatment might have influenced thyroid function. These factors likely contributed to the observed changes in TSH levels.

### 5.2 Treatment of subclinical hypothyroidism in the elderly

Distinguishing between physiological TSH elevation and true hypothyroidism in the elderly is crucial. TSH levels naturally increase with age due to a decline in HPT axis function, leading to higher TSH secretion. Additionally, physiological changes like reduced basal metabolic rate and decreased liver and kidney function may contribute to this phenomenon (38). Hence, the therapeutic benefits of treating SCH in elderly patients may be limited, primarily due to their lower tolerance to L-T4. Initiating treatment without thorough evaluation can pose risks, including hyperthyroidism, which may exacerbate cardiac conditions such as heart failure, myocardial ischemia, and arrhythmias. Additionally, inappropriate treatment can lead to decreased bone density, increasing fracture risk (47). A randomized, double-blind, placebo-controlled trial demonstrated that among patients aged 65 and older with SCH (elevated TSH levels, 4.6–19.9 mIU/L, and FT4 within the reference range), L-T4 treatment offered limited cardiovascular benefits (48). Another randomized, double-blind, placebo-controlled trial included 842 patients aged 65 years or older with SCH, defined by elevated TSH levels (4.6–19.9 mIU/L) with at least two measurements over 3 months to 3 years and FT4 levels within the reference range. The study used forest plots and stratified comparisons based on participants' cardiovascular history and age, analyzing cardiovascular outcomes. Results indicated that L-T4 treatment did not significantly improve cardiovascular outcomes in elderly patients with SCH, regardless of their cardiovascular history (49).

In a study involving 185 adults aged 65 and older with mild SCH, defined by elevated TSH levels (4.60–19.99 mIU/L) with at least two measurements taken 3 months to 3 years apart and FT4 levels within the reference range, patients with recent acute coronary syndrome (within 4 weeks) or NYHA class IV heart failure were excluded. Participants, who had cardiovascular risk factors, received either levothyroxine (initial dose 50 µg daily) or a placebo. TSH levels were measured at least twice, 3 months to 3 years apart, and echocardiography was performed at the trial's end. The results showed no significant differences in cardiac systolic or diastolic function, suggesting that most elderly patients with SCH may not benefit from thyroid hormone therapy (50). Baseline TSH levels were 6.26 ± 1.75 mIU/L in the levothyroxine group and 6.47 ± 2.17 mIU/L in the placebo group, both below 10 mIU/L, indicating a possible physiological basis or stress response to non-thyroidal illnesses. A clinical practice guideline for SCH, which evaluates the annual risk of progression to clinical hypothyroidism at 2%–5%—increasing with positive thyroid peroxidase antibodies and elevated TSH levels—strongly recommends against thyroid hormone treatment for adult patients with SCH, except in special cases (women planning to become pregnant or patients with TSH >20 mIU/L). Studies show that thyroid hormone treatment does not significantly improve quality of life or thyroid-related symptoms and lacks evidence of impact on cardiovascular events and mortality. There is also a potential for harm (51), consistent with patients' views on the lack of benefits. Since SCH is not life-threatening and patients are generally healthy at diagnosis, the potential harms, especially mortality risk, vary based on individual quality of life and comorbidities. This aligns with previous research findings.

### 5.3 Individualized treatment of the elderly population

Individualized treatment is recommended for elderly patients, emphasizing that significant therapeutic benefits from L-T4 are limited to those with true hypothyroidism. A comprehensive assessment of cardiovascular history, comorbidities, osteoporosis risk, and relevant lab tests is essential. Key indicators include the degree of TSH elevation, FT4 levels, and thyroid peroxidase antibody (TPOAb) status. Approximately one-third of elevated TSH levels are transient and may normalize upon retesting, and non-thyroidal interventions should be considered, as some elevations may result from stress responses. Lipid profiles, such as triglycerides and LDL-C, should also be evaluated. For patients with TSH ≥10 mIU/L or ≥20 mIU/L, low-dose L-T4 therapy (25–50 µg daily) may be warranted to mitigate potential cardiovascular risks, with adjustments every 4–6 weeks. Regular follow-up is critical to monitor symptoms (heart rate, blood pressure, fatigue) and prevent overtreatment complications. A low-fat, high-fiber diet is recommended. Dosage adjustments should be patient-specific, ensuring precise and personalized management (52).

## 6 Conclusions

The current research on the association between subclinical hypothyroidism (SCH) and cardiovascular diseases presents conflicting conclusions, primarily due to methodological flaws and misinterpretation of thyroid-stimulating hormone (TSH) levels. Many studies rely solely on TSH as a marker, overlooking the dynamic balance of thyroid hormones, such as changes in the FT3/T4 ratio, and ignoring the confounding effects of non-thyroidal factors like chronic inflammation and aging on TSH levels. For instance, elevated TSH may be an adaptive response to metabolic stress or vascular damage rather than an independent marker of thyroid dysfunction. This “TSH-centric” approach has led to irreproducible findings and even misleading conclusions. Moreover, statistical associations often lack a pathophysiological basis, such as ignoring mechanisms like SERCA2a phosphorylation or PCSK9 regulation, trapping research in a “correlation trap” without elucidating causal relationships. To advance research in this area, methodological innovations are needed. Firstly, multi-omics techniques, such as metabolomics and epigenetics, should be employed to explore the heterogeneity of SCH. Secondly, dynamic assessments of thyroid function, like continuous monitoring of TSH and FT3, should be combined with clinical endpoints such as plaque stability and heart failure hospitalization rates. Lastly, treatment strategies should be reexamined, and personalized intervention trials designed for different subgroups, such as individuals with TSH ≥10 mIU/L and positive TPOAb. Only by moving beyond sole reliance on TSH and integrating pathological physiology with clinical phenotypes can we overcome current research bottlenecks and provide evidence-based guidelines for SCH management.

## References

[1] Jonklaas J Bianco AC Bauer AJ Burman KD Cappola AR Celi FS Guidelines for the treatment of hypothyroidism: prepared by the American thyroid association task force on thyroid hormone replacement. Thyroid. (2014) 24(12):1670–751. 10.1089/thy.2014.0028 25266247 PMC4267409

[2] Sue LY Leung AM. Levothyroxine for the treatment of subclinical hypothyroidism and cardiovascular disease. Front Endocrinol (Lausanne). (2020) 11:591588. 10.3389/fendo.2020.591588 33193104 PMC7609906

[3] Stott DJ Rodondi N Kearney PM Ford I Westendorp RGJ Mooijaart SP Thyroid hormone therapy for older adults with subclinical hypothyroidism. N Engl J Med. (2017) 376(26):2534–44. 10.1056/NEJMoa1603825 28402245

[4] Patrizio A Ferrari SM Elia G Ragusa F Balestri E Botrini C Hypothyroidism and metabolic cardiovascular disease. Front Endocrinol (Lausanne). (2024) 15:1408684. 10.3389/fendo.2024.1408684 38887272 PMC11180764

[5] Brent GA. Mechanisms of thyroid hormone action. J Clin Invest. (2012) 122(9):3035–43. 10.1172/JCI60047 22945636 PMC3433956

[6] Vanderpump MP. The epidemiology of thyroid disease. Br Med Bull. (2011) 99:39–51. 10.1093/bmb/ldr030 21893493

[7] Roelfsema F Veldhuis JD. Thyrotropin secretion patterns in health and disease. Endocr Rev. (2013) 34(5):619–57. 10.1210/er.2012-1076 23575764

[8] Dietrich JW Hoermann R Midgley JEM Bergen F Müller P. The two faces of Janus: why thyrotropin as a cardiovascular risk factor may be an ambiguous target. Front Endocrinol (Lausanne). (2020) 11:542710. 10.3389/fendo.2020.542710 33193077 PMC7649136

[9] Müller P Leow MK Dietrich JW. Minor perturbations of thyroid homeostasis and major cardiovascular endpoints-physiological mechanisms and clinical evidence. Front Cardiovasc Med. (2022) 9:942971. 10.3389/fcvm.2022.942971 36046184 PMC9420854

[10] Tian L Song Y Xing M Zhang W Ning G Li X A novel role for thyroid-stimulating hormone: up-regulation of hepatic 3-hydroxy-3-methyl-glutaryl-coenzyme A reductase expression through the cyclic adenosine monophosphate/protein kinase A/cyclic adenosine monophosphate-responsive element binding protein pathway. Hepatology. (2010) 52(4):1401–9. 10.1002/hep.23800 20648556

[11] Delitala AP Fanciulli G Maioli M Delitala G. Subclinical hypothyroidism, lipid metabolism and cardiovascular disease. Eur J Intern Med. (2017) 38:17–24. 10.1016/j.ejim.2016.12.015 28040402

[12] Zhang X Song Y Feng M Zhou X Lu Y Gao L Thyroid-stimulating hormone decreases HMG-CoA reductase phosphorylation via AMP-activated protein kinase in the liver. J Lipid Res. (2015) 56(5):963–71. 10.1194/jlr.M047654 25713102 PMC4409286

[13] Fazaeli M Khoshdel A Shafiepour M Rohban M. The influence of subclinical hypothyroidism on serum lipid profile, PCSK9 levels and CD36 expression on monocytes. Diabetes Metab Syndr. (2019) 13(1):312–6. 10.1016/j.dsx.2018.08.021 30641718

[14] Gong Y Ma Y Ye Z Fu Z Yang P Gao B Thyroid stimulating hormone exhibits the impact on LDLR/LDL-c via up-regulating hepatic PCSK9 expression. Metab Clin Exp. (2017) 76:32–41. 10.1016/j.metabol.2017.07.006 28987238

[15] Harrar S Mhirig I El Alaoui Boufares Y Bouchehboun A Bounani F Aboulmakarim S. Lipid profile perturbations associated with subclinical hypothyroidism: a descriptive study. Cureus. (2024) 16(4):e58181. 10.7759/cureus.58181 38741822 PMC11089583

[16] Mavromati M Jornayvaz FR. Hypothyroidism-Associated dyslipidemia: potential molecular mechanisms leading to NAFLD. Int J Mol Sci. (2021) 22(23):12797. 10.3390/ijms222312797 34884625 PMC8657790

[17] Yildirim AM Koca AO Beyan E Dogan O Karakaya S Aksoz Z Association of serum proprotein convertase subtilisin/kexin type 9 (PCSK9) level with thyroid function disorders. Eur Rev Med Pharmacol Sci. (2021) 25(17):5511–7. 10.26355/eurrev_202109_26662 34533801

[18] Janovsky CCPS Generoso G Goulart AC Santos RD Blaha MJ Jones S Differences in HDL particle size in the presence of subclinical thyroid dysfunctions: the ELSA-Brasil study. Atherosclerosis. (2020) 312:60–5. 10.1016/j.atherosclerosis.2020.08.021 32977122

[19] Desideri G Bocale R D'Amore A Necozione S Boscherini M Carnassale G Replacement therapy with levothyroxine modulates platelet activation in recent-onset post-thyroidectomy subclinical hypothyroidism. Nutr Metab Cardiovasc Dis. (2017) 27(10):896–901. 10.1016/j.numecd.2017.07.002 28964662

[20] Zha K Zuo C Wang A Zhang B Zhang Y Wang B LDL in patients with subclinical hypothyroidism shows increased lipid peroxidation. Lipids Health Dis. (2015) 14:95. 10.1186/s12944-015-0092-4 26302822 PMC4548906

[21] Saric MS Jurasic MJ Budincevic H Milosevic M Kranjcec B Kovacic S The role of thyroid hormones in carotid arterial wall remodeling in women. Rom J Intern Med. (2022) 60(1):24–33. 10.2478/rjim-2021-0028 34303321

[22] de Miranda ÉJ P Bittencourt MS Pereira AC Goulart AC Santos IS Lotufo PA Subclinical hypothyroidism is associated with higher carotid intima-media thickness in cross-sectional analysis of the Brazilian longitudinal study of adult health (ELSA-Brasil). Nutr Metab Cardiovasc Dis. (2016) 26(10):915–21. 10.1016/j.numecd.2016.06.005 27389191

[23] Yao K Zhao T Zeng L Yang J Liu Y He Q Non-invasive markers of cardiovascular risk in patients with subclinical hypothyroidism: a systematic review and meta-analysis of 27 case control studies. Sci Rep. (2018) 8(1):4579. 10.1038/s41598-018-22897-3 29545561 PMC5854616

[24] Isailă OM Stoian VE Fulga I Piraianu AI Hostiuc S. The relationship between subclinical hypothyroidism and carotid intima-media thickness as a potential marker of cardiovascular risk: a systematic review and a meta-analysis. J Cardiovasc Dev Dis. (2024) 11(4):98. 10.3390/jcdd11040098 38667716 PMC11049994

[25] Floriani C Gencer B Collet TH Rodondi N. Subclinical thyroid dysfunction and cardiovascular diseases: 2016 update. Eur Heart J. (2018) 39(7):503–7. 10.1093/eurheartj/ehx050 28329380

[26] Dong J Gao C Liu J Cao Y Tian L. TSH inhibits SERCA2a and the PKA/PLN pathway in rat cardiomyocytes. Oncotarget. (2016) 7(26):39207–15. 10.18632/oncotarget.9393 27206677 PMC5129926

[27] Yao Z Gao X Liu M Chen Z Yang N Jia YM Diffuse myocardial injuries are present in subclinical hypothyroidism: a clinical study using myocardial T1-mapping quantification. Sci Rep. (2018) 8(1):4999. 10.1038/s41598-018-22970-x 29567964 PMC5864753

[28] Bernardi S Grillo A Antonello RM Cola MF Dobrinja C Fabris B Meta-analysis on the association between thyroid hormone disorders and arterial stiffness. J Endocr Soc. (2022) 6(4):bvac016. 10.1210/jendso/bvac016 35284772 PMC8907416

[29] Gao F. Variation tendency of coagulation parameters in different hypothyroidism stages. Acta Endocrinol (Bucharest). (2016) 12(4):450–4. 10.4183/aeb.2016.450 PMC6535246 31149130

[30] Elbers LPB Fliers E Cannegieter SC. The influence of thyroid function on the coagulation system and its clinical consequences. J Thromb Haemost. (2018) 16(4):634–45. 10.1111/jth.13970 29573126

[31] Gluvic ZM Obradovic MM Sudar-Milovanovic EM Zafirovic SS Radak DJ Essack MM Regulation of nitric oxide production in hypothyroidism. Biomed Pharmacother. (2020) 124:109881. 10.1016/j.biopha.2020.109881 31986413

[32] Su X Peng H Chen X Wu X Wang B. Hyperlipidemia and hypothyroidism. Clin Chim Acta. (2022) 527:61–70. 10.1016/j.cca.2022.01.006 35038435

[33] Gao C Li T Liu J Guo Q Tian L. Endothelial functioning and hemodynamic parameters in rats with subclinical hypothyroid and the effects of thyroxine replacement. PLoS One. (2015) 10(7):e0131776. 10.1371/journal.pone.0131776 26158620 PMC4497722

[34] Jabbar A Pingitore A Pearce SH Zaman A Iervasi G Razvi S. Thyroid hormones and cardiovascular disease. Nat Rev Cardiol. (2017) 14(1):39–55. 10.1038/nrcardio.2016.174 27811932

[35] Moon S Kim MJ Yu JM Yoo HJ Park YJ. Subclinical hypothyroidism and the risk of cardiovascular disease and all-cause mortality: a meta-analysis of prospective cohort studies. Thyroid. (2018) 28(9):1101–10. 10.1089/thy.2017.0414 29978767

[36] Fisher AJ Medaglia JD Jeronimus BF. Lack of group-to-individual generalizability is a threat to human subjects research. Proc Natl Acad Sci U S A. (2018) 115(27):E6106–15. 10.1073/pnas.1711978115 29915059 PMC6142277

[37] Brenowitz WD Han F Kukull WA Nelson PT. Treated hypothyroidism is associated with cerebrovascular disease but not Alzheimer’s disease pathology in older adults. Neurobiol Aging. (2018) 62:64–71. 10.1016/j.neurobiolaging.2017.10.004 29107848 PMC5743774

[38] Biondi B Cappola AR Cooper DS. Subclinical hypothyroidism: a review. JAMA. (2019) 322(2):153–60. 10.1001/jama.2019.9052 31287527

[39] Rodondi N den Elzen WP Bauer DC Cappola AR Razvi S Walsh JP Subclinical hypothyroidism and the risk of coronary heart disease and mortality. JAMA. (2010) 304(12):1365–74. 10.1001/jama.2010.1361 20858880 PMC3923470

[40] Lawton RI Sabatini BL Hochbaum DR. Longevity, demographic characteristics, and socio-economic status are linked to triiodothyronine levels in the general population. Proc Natl Acad Sci U S A. (2024) 121(2):e2308652121. 10.1073/pnas.2308652121 38175866 PMC10786306

[41] Baumgartner C da Costa BR Collet TH Feller M Floriani C Bauer DC Thyroid function within the normal range, subclinical hypothyroidism, and the risk of atrial fibrillation. Circulation. (2017) 136(22):2100–16. 10.1161/CIRCULATIONAHA.117.028753 29061566 PMC5705446

[42] Zhao T Chen B Zhou Y Wang X Zhang Y Wang H Effect of levothyroxine on the progression of carotid intima-media thickness in subclinical hypothyroidism patients: a meta-analysis. BMJ Open. (2017) 7(10):e016053. 10.1136/bmjopen-2017-016053 29061604 PMC5665330

[43] Razvi S. Novel uses of thyroid hormones in cardiovascular conditions. Endocrine. (2019) 66(1):115–23. 10.1007/s12020-019-02050-4 31617169 PMC6794474

[44] Fitzgerald SP Bean NG Falhammar H Tuke J. Clinical parameters are more likely to be associated with thyroid hormone levels than with thyrotropin levels: a systematic review and meta-analysis. Thyroid. (2020) 30(12):1695–709. 10.1089/thy.2019.0535 32349628 PMC7757573

[45] Wang W Zhang X Gao J Meng X Wang J Zhang K Effects of levothyroxine in subclinical hypothyroidism and heart failure with reduced ejection fraction: an open-label randomized trial. Cell Rep Med. (2024) 5(4):101473. 10.1016/j.xcrm.2024.101473 38537636 PMC11031377

[46] Jabbar A Ingoe L Junejo S Carey P Addison C Thomas H Effect of levothyroxine on left ventricular ejection fraction in patients with subclinical hypothyroidism and acute myocardial infarction: a randomized clinical trial. JAMA. (2020) 324(3):249–58. 10.1001/jama.2020.9389 32692386 PMC12068826

[47] Feldt-Rasmussen U Effraimidis G Bliddal S Klose M. Risks of suboptimal and excessive thyroid hormone replacement across ages. J Endocrinol Invest. (2024) 47(5):1083–90. 10.1007/s40618-023-02229-7 38015369 PMC11035408

[48] Mooijaart SP Du Puy RS Stott DJ Kearney PM Rodondi N Westendorp RGJ Association between levothyroxine treatment and thyroid-related symptoms among adults aged 80 years and older with subclinical hypothyroidism. JAMA. (2019) 322(20):1977–86. 10.1001/jama.2019.17274 31664429 PMC6822162

[49] Zijlstra LE Jukema JW Westendorp RGJ Du Puy RS Poortvliet RKE Kearney PM Levothyroxine treatment and cardiovascular outcomes in older people with subclinical hypothyroidism: pooled individual results of two randomised controlled trials. Front Endocrinol (Lausanne). (2021) 12:674841. 10.3389/fendo.2021.674841 34093444 PMC8173189

[50] Gencer B Moutzouri E Blum MR Feller M Collet TH Delgiovane C The impact of levothyroxine on cardiac function in older adults with mild subclinical hypothyroidism: a randomized clinical trial. Am J Med. (2020) 133(7):848–56.e5. 10.1016/j.amjmed.2020.01.018 32171774

[51] Bekkering GE Agoritsas T Lytvyn L Heen AF Feller M Moutzouri E Thyroid hormones treatment for subclinical hypothyroidism: a clinical practice guideline. BMJ. (2019) 365:l2006. 10.1136/bmj.l2006 31088853

[52] Chrysant SG. The current debate over treatment of subclinical hypothyroidism to prevent cardiovascular complications. Int J Clin Pract. (2020) 74(7):e13499. 10.1111/ijcp.13499 32159256

